# Novel Fingertip Image-Based Heart Rate Detection Methods for a Smartphone

**DOI:** 10.3390/s17020358

**Published:** 2017-02-12

**Authors:** Rifat Zaman, Chae Ho Cho, Konrad Hartmann-Vaccarezza, Tra Nguyen Phan, Gwonchan Yoon, Jo Woon Chong

**Affiliations:** 1 Department of Electrical and Computer Engineering, Texas Tech University, Lubbock, TX 79409, USA; rifat.zaman@ttu.edu (R.Z.); backjoe@gmail.com (C.H.C.); tra.phan@ttu.edu (T.N.P.); gwonchanyoon@gmail.com (G.Y.); 2 Instituto de Ingenieria Biológica y Médica, Pontificia Universidad Católica de Chile, Santiago 7820436, Chile; kthartmann@uc.cl

**Keywords:** heart rate detection, health monitoring, smartphone discipline

## Abstract

We hypothesize that our fingertip image-based heart rate detection methods using smartphone reliably detect the heart rhythm and rate of subjects. We propose fingertip curve line movement-based and fingertip image intensity-based detection methods, which both use the movement of successive fingertip images obtained from smartphone cameras. To investigate the performance of the proposed methods, heart rhythm and rate of the proposed methods are compared to those of the conventional method, which is based on average image pixel intensity. Using a smartphone, we collected 120 s pulsatile time series data from each recruited subject. The results show that the proposed fingertip curve line movement-based method detects heart rate with a maximum deviation of 0.0832 Hz and 0.124 Hz using time- and frequency-domain based estimation, respectively, compared to the conventional method. Moreover, another proposed fingertip image intensity-based method detects heart rate with a maximum deviation of 0.125 Hz and 0.03 Hz using time- and frequency-based estimation, respectively.

## 1. Introduction

Recently, smartphones have been widely spread and used in our daily life. Moreover, smartphones have rapidly evolved in terms of hardware, software, and application. For example, central processing unit (CPU), memory, and battery performance of smartphones are enhanced fast; embedded sensors got diverse; and various types of applications with different operating systems (OSs) are actively developed for smartphones. As a result, smartphone applications are not only assisting office and personal work but also providing entertaining contents/games. Especially, health monitoring smartphone applications has gained much attention due to users’ increased interest in point-of-care health technologies [1].

An important and promising health monitoring smartphone application is heart rhythm monitoring application since heart rhythm monitoring provides information about heart healthiness as well as vital sign information. For example, heart rhythm monitoring is used to detect if a subject has atrial fibrillation (AF) since AF gives irregular heart rhythms. AF is the most common arrhythmia and can lead to serious complications such as stroke and heart failure [2,3,4].

Nowadays, smartphones are considered to be effective for heart rhythm monitoring. Specifically, heart rhythm monitoring based on smartphone camera recordings of fingertips are shown to give heart rhythms in an accurate way since there is vibration and blood flow through the pulmonary vessels beneath fingertips [5,6,7,8,9]. In this paper, we propose novel heart rate detection methods for iPhone 4S/5S/6S, which can detect heart rate using the changes of fingertip images recorded from a smartphone’s video camera. To detect changes of fingertip images, we considered two different approaches: (1) *fingertip curve line movement detection*; and (2) *fingertip intensity movement detection*. To increase the detectability of heart rate in fingertip curve movement detection, we adopted bit arrangement, edge detection, and smoothing techniques. The edge detection [10,11,12,13] and smoothing techniques [14,15,16] have been widely used in detecting objects and removing noises in digital image processing. In this study, we evaluate our heart rate detection algorithms’ performance with data from healthy subjects who were recruited after consenting for our study at Texas Tech University (TTU) (Lubbock, TX, USA). The rest of this paper is organized as follows: Section 2 describes experimental protocol and preprocessing in our research; Section 3 explains two different proposed approaches which are both based on fingertip images changes; Section 4 evaluates the performance of the proposed approaches on test data; and Section 5 concludes this paper.

## 2. Materials

### 2.1. Experimental Protocol

**Figure 1:**
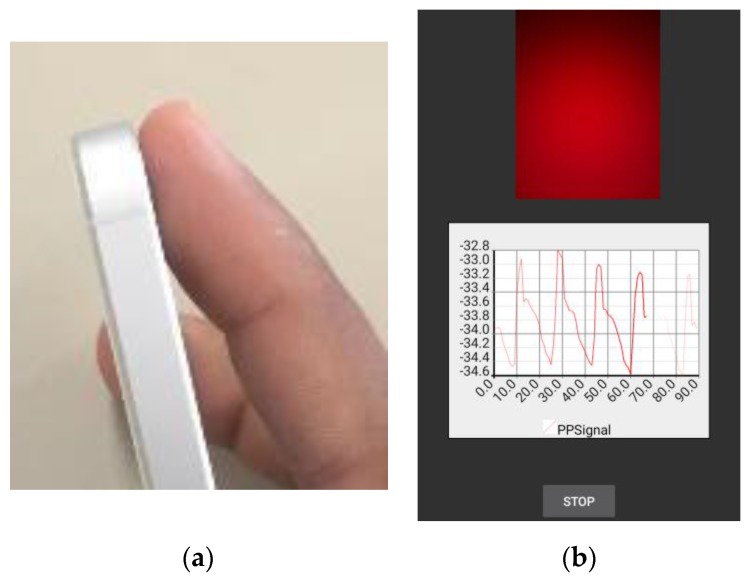
Our developed smartphone-based heart rate detection application: (**a**) fingertip placement; and (**b**) graphic user interface (GUI) of our developed application.

We obtain video camera recording of fingertip data from volunteering subjects using iPhone 4/5/6. We recruited volunteering participants between 18 and 80 years old at TTU without diagnosed cardiovascular and tremor problems that limit keeping hands still during fingertip video camera recording measurement. Here, the recruited volunteering subjects are healthy. Our collection data protocol was approved by the Institutional Review Boards of TTU (#2016-764). The subjects all gave signed consent forms. The subjects are asked to sit and place their fingertips on a camera lens as shown in Figure 1a. We start our smartphone application after guiding the subject not to move their hands with maintaining his/her fingertip placed on a lens as shown in Figure 1a. Our developed algorithm records the subject’s fingertip images for two minutes. The developed smartphone application turns off in an automatic way after this two minutes of recordings. We downloaded this recorded data from the smartphone to an offline server, and performed signal processing and data analysis using MATLAB in the server.

### 2.2. Preprocessing

Our developed heart rate detection method was implemented as a smartphone application. The graphical user interface (GUI) of our developed application is shown in Figure 1b. The smartphone application records a sequence of fingertip images from the smartphone video camera showing its average pixel intensity values in real-time. The development platform of iPhone 4/5/6 is iOS. iPhone 4/5/6 have enough central processing unit (CPU) speed/random access memory (RAM) size of 800 MHz/512 MB, 1.2 GHz/1 GB, and 1.38 GHz/1 GB, respectively, to handle smartphone camera recording operation and preprocess/process a successive sequence of fingertip images in real-time. The video cameras give sampling rates/image resolutions of 30 frames per second (fps)/1280 × 720 pixels for iPhone 4, and 30 fps/1920 × 1080 pixels for iPhone 5/6. To reduce computational complexity, we use upper left part (640 × 480 pixels) of the recorded images since this upper left part was shown to give better estimate of heart rhythm change compared the other parts [5].

## 3. Methods

We propose two heart rate detection methods based on successive fingertip images taken by smartphone video cameras: fingertip curve line movement-based detection and fingertip image-intensity based detection methods. The details of these proposed methods are described in Section 3.1 and Section 3.2, respectively.

### 3.1. Fingertip Curve Line Movement-Based Detection

**Figure 2:**
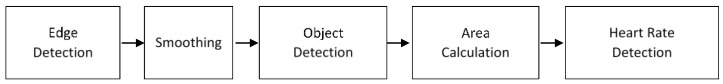
Procedure of the fingertip curve line movement-based detection method.

Fingertip curve line movement-based heart rate detection method detects the heart rate from the movement (or change) of fingertip curves in successive recorded fingertip images. This proposed method consists of a sequence of procedures shown in Figure 2. Along this sequence of procedures, the image format is converted when needed in order to either increase a camera’s sensitivity to the fingertip curve movement or reduce the computational complexity. Specifically, the image format is converted following these three steps: (1) recorded raw images are converted into RGB 565 images where each pixel is represented by five red, six green, and five blue bits; (2) RGB 565 images are converted to RGB 888 format with bit arrangement, compressed into red images by extracting only eight red bits from RGB 888 images; and (3) RGB 888 images are converted into gray-scale images which are obtained by directly assigning eight bits of intensity values in red images into those in grayscale images [17].

In a sequence of the gray-scale images, edge detection and smoothing techniques are applied in order to detect major fingertip curve lines with filtering out low and high frequency components in the images, and each technique is described in Section 3.1.1 and Section 3.1.2.

#### 3.1.1. Edge Detection

**Figure 3:**
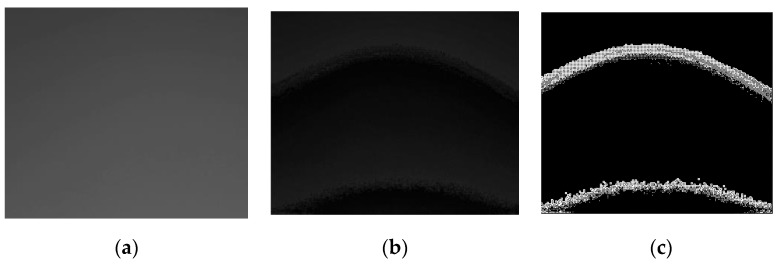
An example of the edge detection procedure on a fingertip image obtained from a smartphone video camera: (**a**) greyscale image in YUV raw image; (**b**) preprocessed image; and (**c**) the edge detected image.

**Figure 4:**
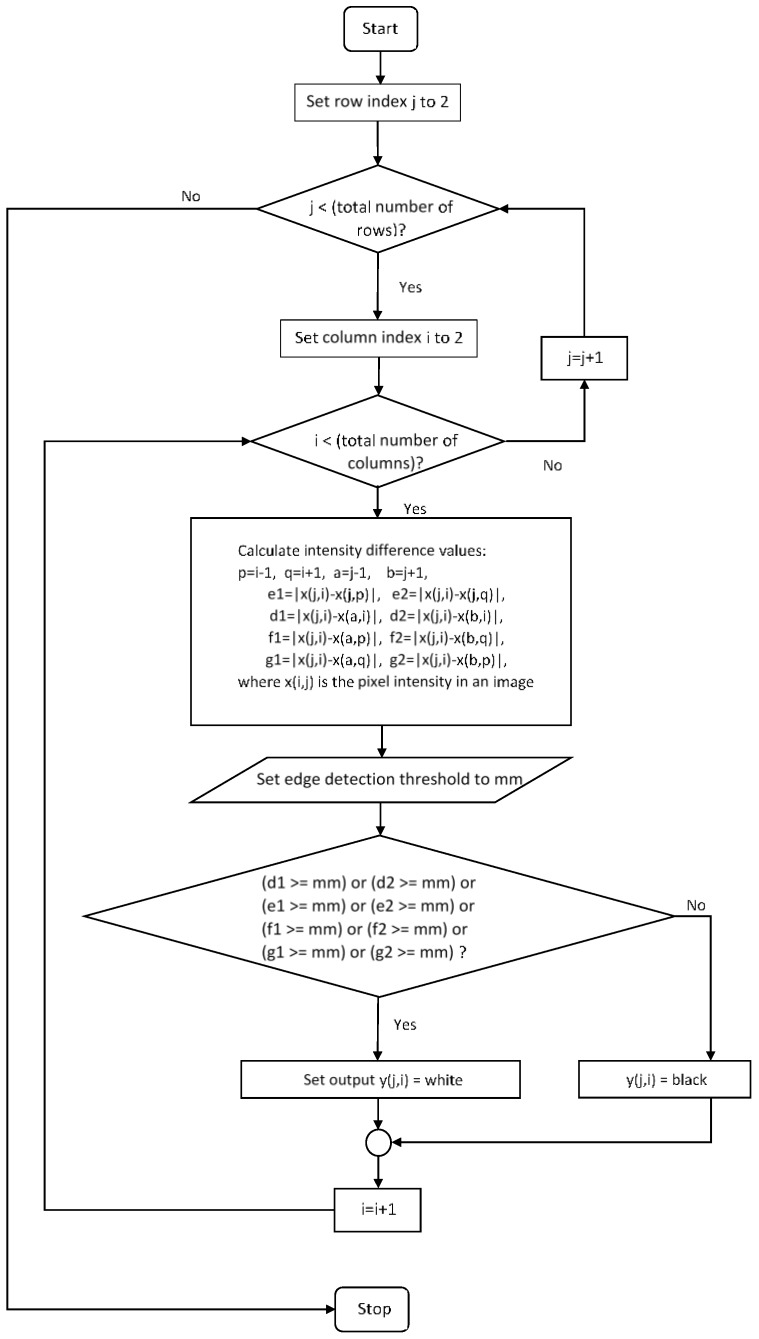
Flow chart of our proposed edge detection procedure.

An edge in image processing is defined to be a set of points (or a segment) where image brightness (or intensity) changes abruptly [10,11,12,13,14,15,16]. In the proposed method, the edge detection procedure finds these edges (or fingertip curve line) based on the intensity value *x*(*i, j*) of pixel *(i, j)*, where *i* and *j* are *x*- and *y*-axis position values in the grayscale image, respectively. Denoting the maximum intensity value difference between neighboring pixels by $\Delta x{\left(i,j\right)}_{\max}=\max\left\{x\left(i,j\right)-x\left(k,l\right)\right\}$ where k∈{i−1,i, i+1} and l∈{j−1,j, j+1}, $\Delta x{\left(i,j\right)}_{\max}$ values at the edges are expected to be larger than those at non-edge points. Here, edge detection output y is derived by mapping $\Delta x{\left(i,j\right)}_{\max}$ to y(i,j) where y(i,j) is 0 (white) when $\Delta x{\left(i,j\right)}_{\max}>A_{\mathrm{th}}$ and y(i,j) is 1 (black) when $\Delta x{\left(i,j\right)}_{\max}<A_{\mathrm{th}}$, where $A_{\mathrm{th}}$ is a pre-defined threshold value for the Δx(i,j). The Y-image of raw (or YUV) image (Figure 3a) is preprocessed (Figure 3b) as described in Section 3.1, and the edges in Figure 3b are more visible compared to those in Figure 3a. The edge detection procedure changes greyscale input image (Figure 3a) into binary output image (Figure 3c). The pseudocode of this edge detection procedure is shown in Figure 4.

#### 3.1.2. Smoothing

**Figure 5:**
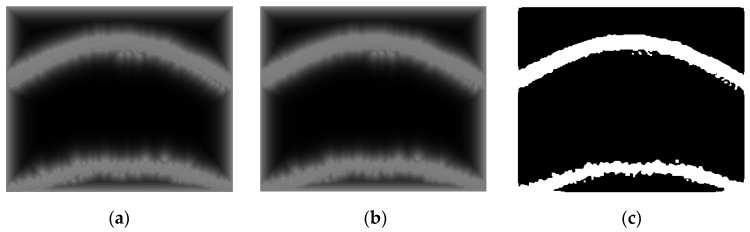
An example of the smoothing procedure on a fingertip image obtained from a smartphone video camera: (**a**) after distance transform; (**b**) after diffusion; and (**c**) after binarization.

Smoothing is a procedure of capturing significant patterns in an image while leaving isolated (or fine) patterns out. Since the edge detection procedure can detect both isolated and adjoined noticeable points, as shown in Figure 5a, smoothing is needed to detect only adjoined noticeable points, e.g., fingertip curves. In the smoothing procedure, individual (or isolated) points coming from noise are decreased while the points near adjoining points are increased leading to a smoother line (or segment).

We adopt the anisotropic diffusion-based smoothing method, which consists of distance transform, anisotropic smoothing, and binarization [18,19]. In the distance transform step, the binary image having edges obtained in the edge detection procedure (see Section 3.1.1) is transformed into a distance-transformed grayscale image. This step facilitates the next anisotropic smoothing step by diffusing discrete white points (detected noticeable points) into their neighboring regions as shown in Figure 5a (edge detected is given in Figure 3c). The anisotropic smoothing step is a main step which performs smoothing in the grayscale image by giving more weight (diffusion) to the directions (anisotropic) of the distance transformed images [18,19]. The grayscale image obtained by this anisotropic approach is shown in Figure 5b. The binarization step with a pre-defined threshold value finalizes the smoothing procedure by converting smoothed grayscale images into black and white images, as shown in Figure 5c.

#### 3.1.3. Fingertip Curved Region Detection

Fingertip curved regions are detected in successive smoothed images to detect the movements (or changes) of the curved regions, which are considered to reflect heart rhythms. Here, a fingertip curved region is defined as a region closed by the surrounding curves obtained in Section 3.1.2, or image boundaries in smoothed images. The procedure of this fingertip curved region detection step can be explained in a sequential way: (1) initial phase *(i*_frame_ *=* 0*)* and (2) remaining phase *(i*_frame_ *>* 0), where *i*_frame_ denotes the index of the recorded image frame taken by a smartphone video camera. To facilitate this region detection procedure, we set the color of the pixels on image boundaries (right, left, up, down) to black.

**Figure 6:**
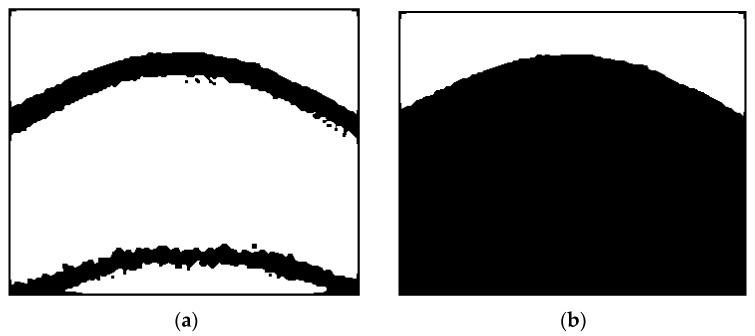
An example of the fingertip curved region detection procedure on a fingertip image obtained from a smartphone video camera: (**a**) non-trivial three regions (white) are found in an inverting smoothed image and (**b**) one representative region having the largest *d_avg_* is found.

In the initial phase, the regions having average distance *d_avg_*, from their centroids, larger than a predefined threshold value *D*_th_ are found among the detected curved regions, and are called non-trivial regions. Assuming that the movements of these non-trivial regions are all reflecting heart rhythms, we choose only one representative region, which has the largest average distance *d_avg_* among the non-trivial regions bounded by either the upper or lower smoothed curved line, to reduce computational complexity in further procedures of Section 3.1.4. For example, three non-trivial (white) regions, after inverting the smoothed image of Figure 5c, are separated as shown in Figure 6a. After setting four boundary edges to black, we find two regions which have upper or lower smoothed curved lines. Between the two found regions, the upper region is chosen to be a representative region, as shown in Figure 6b, since it has a larger average distance *d_avg_* between the upper and lower regions which are bounded by either the lower or upper smoothed curved lines, respectively. Finally, the number of pixels in the representative region is counted and logged. In the remaining phase, representative regions in successively recorded fingertip images are detected, and the number of pixels inside the representative region are logged.

#### 3.1.4. Heart Rhythm and Rate Detection

As mentioned in Section 3.1.3, the area of a region is calculated by counting the number of pixels within the representative region. Heart rhythm is detected based on this area changes of the representative region in this proposed method. The area of a region is logged in the previous step (see Section 3.1.3). Heart rate is detected in two ways: (1) *peak detection (time-domain)*; and *(*2) *dominant frequency detection (frequency-domain)*. The time-domain approach estimates a heart rate by inversing the median value of a set of successive peak-to-peak intervals while the frequency-domain approach estimates by Welch’s power spectrum estimation method within 0.5 Hz to 2 Hz [20].

### 3.2. Fingertip Image Intensity-Based Detection

**Figure 7:**
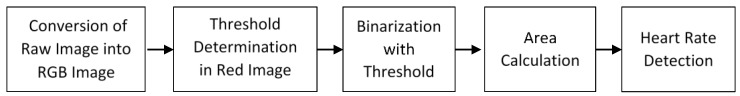
Procedure of the fingertip image intensity-based detection method.

**Figure 8:**
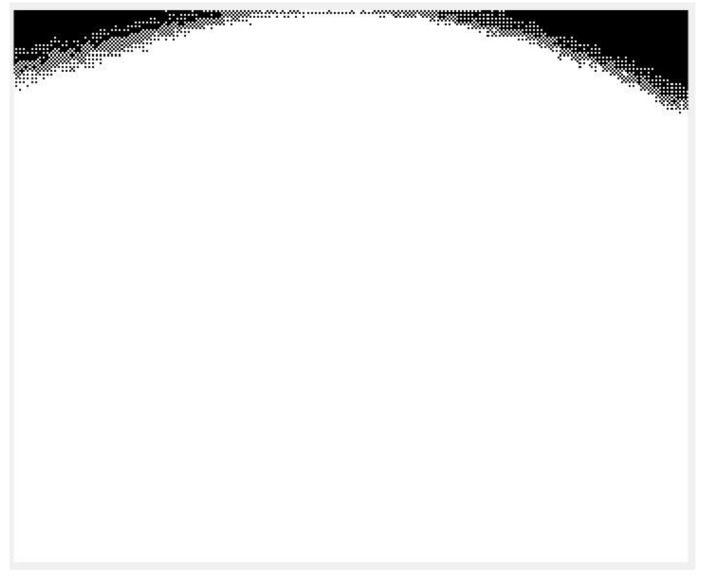
An example of fingertip image intensity-based detection results obtained from a smartphone video camera.

The fingertip image intensity-based heart rate detection method detects the heart rate from the movement (or change) of fingertip images, binarized based on intensities, in successive recorded fingertip images. The flowchart of this proposed method is shown in Figure 7. Along this sequence of procedures, (1) raw images obtained from a smartphone camera are converted into RGB images; and (2) the red signals in the RGB images are converted into binary images. Specifically, the latter binarization step is performed with the initial median intensity value *R*_intensity_, which is defined as the median intensity value in an initial image frame. Denoting row and column indices in an image by *i* and *j*, respectively, the binarization step compares the intensity values *r*(*i*, *j*) of each pixel in the initial image frame to *R*_intensity_. If *r*(*i*, *j*) exceeds *R*_intensity_, then the value *s*(*i*, *j*) of the output binary image is 1 (white). Otherwise, *s*(*i*, *j*) becomes 0 (black). Figure 8 gives an example of this binarization result. 

With this white region as the representative fingertip curved region, heart rhythm and rate detection is performed by the same procedure described in Section 3.1.4.

## 4. Results

We evaluated the performance of the proposed heart rate detection ratealgorithms for the iPhone data. A total of 120 s of data is collected from each subject. We compared our heart detection algorithm to the previous arrhythmia discrimination algorithms [5], which detects heart rate from the pulsatile time series data obtained by averaging the pixel intensities of successive images. Specifically, the threshold value for anisotropic diffusion was set to −2. As performance metrics, we considered the estimated pulsatile heart rhythm and rate.

**Figure 9:**
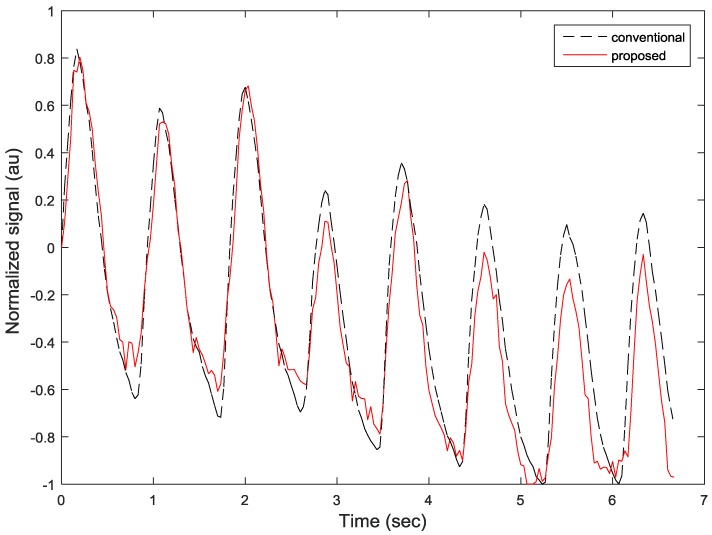
Estimated heart rhythms of the proposed fingertip curve line movement-based algorithm and the conventional average intensity-based algorithm.

**Figure 10:**
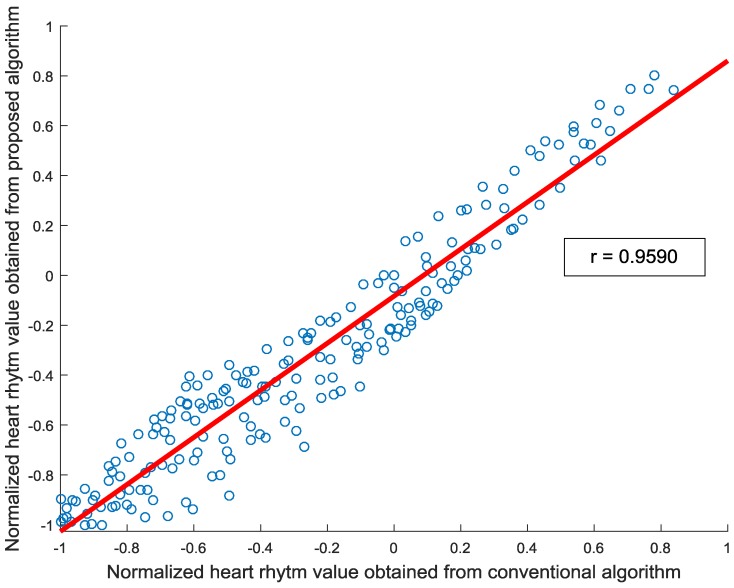
An example of the correlation between heart rhythms obtained from the proposed fingertip curve line movement-based and the conventional heart rate detection methods for subject 2. Here, the correlation line is denoted by the red solid line and the correlation index is 0.9590.

Figure 9 compares the estimated heart rhythms of the proposed fingertip curve line movement-based algorithm and the conventional average intensity-based algorithm, and shows that they are similar each other. We performed a correlation test on clean signals to evaluate the correlation between the proposed and conventional algorithms. The correlation coefficient values between peak locations obtained by the proposed fingertip curve line movement-based and conventional heart rate detection methods are 0.9999 for subjects 1, 2, 3, 4, 6, 7, 8, and 9; 0.9998 for subject 5; and 0.9996 for subject 10. These high correlation coefficient values indicate that the peak locations obtained from the proposed and conventional algorithms are highly correlated to each other. Inversely, the correlation coefficient values between sampled heart rhythm values of the proposed and conventional methods are relatively lower compared to the correlation coefficient values of the peak locations above since the two methods measure rhythms in different ways. For example, the correlation coefficient value between sampled heart rhythm values is 0.9590 for subject 2, as shown in Figure 10.

**Figure 11:**
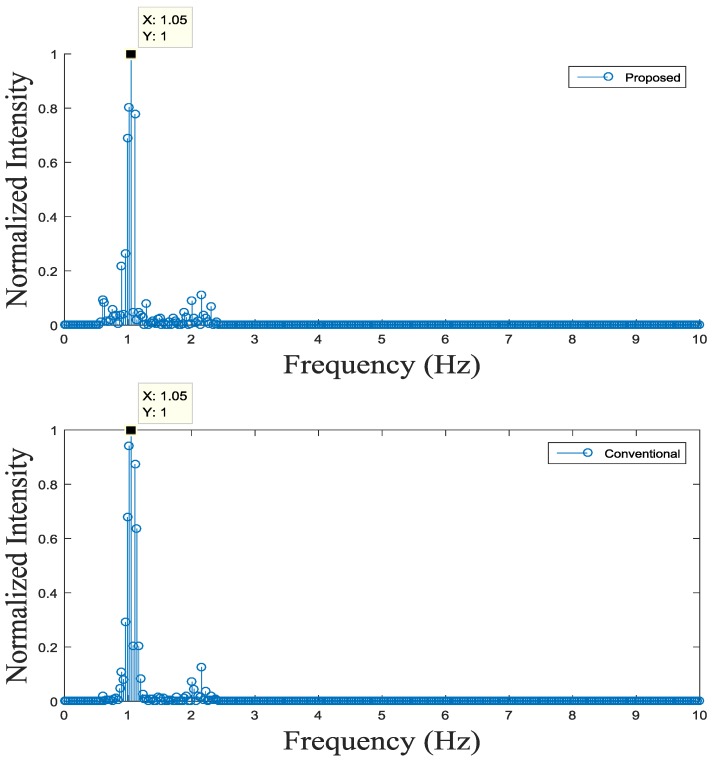
An example of the heart rate estimation of the proposed fingertip curve line movement-based and the conventional heart rate detection methods (frequency-domain estimation).

We estimate heart rate from time series signals, obtained in Section 4.1, using time- or frequency-domain methods, as explained in Section 3.1.4. Specifically, in frequency-domain method, heart rate is estimated by deriving dominant frequency within 0.5 Hz–2 Hz in Welch’s power spectrum of the time series signal. Figure 11 shows the Welch’s power spectrum of the time series signals obtained from (a) the proposed fingertip curve line movement detection; and (b) the conventional method. The proposed detection methods give more power of the heart rate frequency component than the conventional method, while the proposed and conventional methods give similar dominant frequencies. Estimated heart rates of our proposed and conventional algorithms from 10 subjects are shown in Table 1. Specifically, the maximum deviation is 0.0832 Hz using the time-based estimation method, while they have the maximum deviation of 0.124 Hz using the frequency-based estimation method.

### Fingertip Image Intensity-Based Method

**Figure 12:**
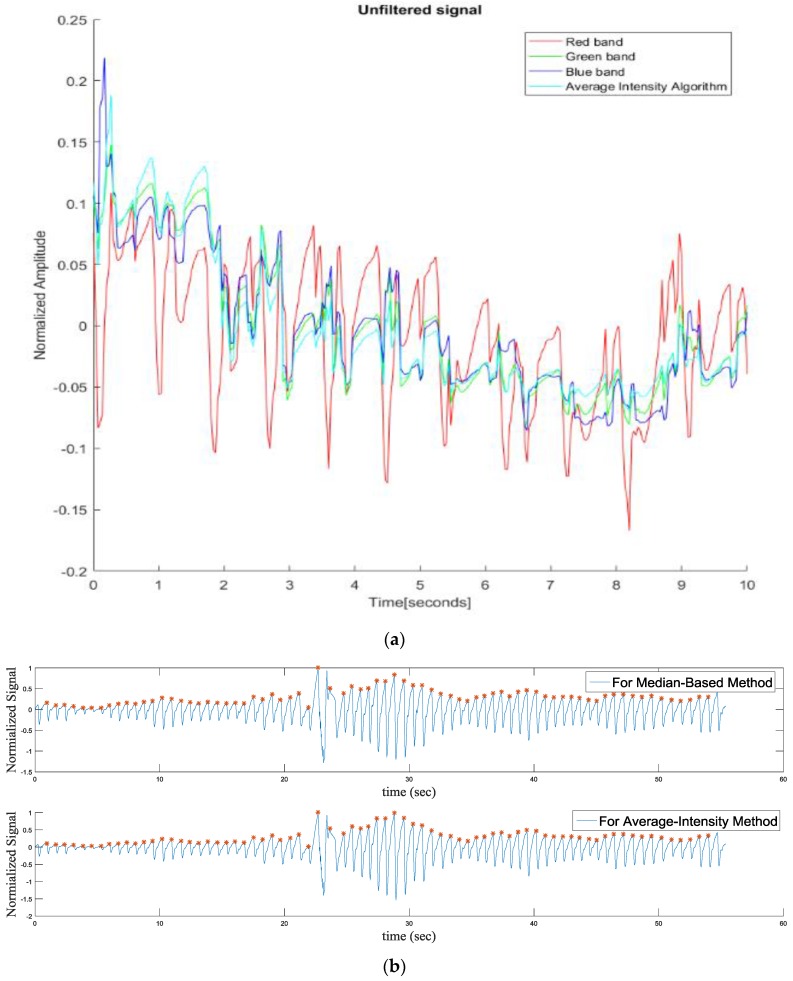
Heart rhythms obtained by the proposed (median-based) and conventional methods: (**a**) heart rhythms from the conventional RGB band-based method and the proposed method; and (**b**) peak detection results from the conventional red band-based method and the proposed method.

**Figure 13:**
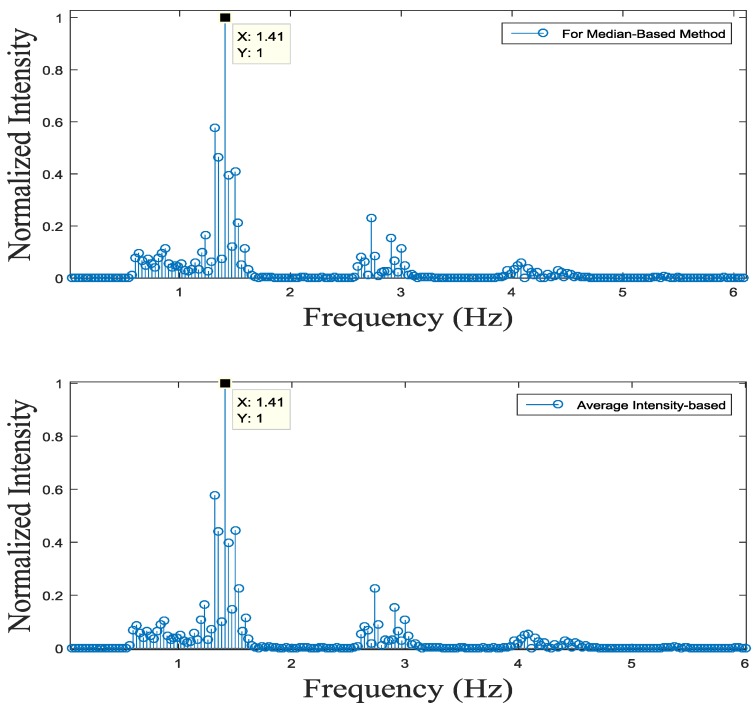
Frequency-domain analysis of heart rhythms obtained by the proposed (median-based) and conventional methods.

Figure 12a compares heart rhythm estimations from our proposed fingertip image intensity-based and conventional methods. As shown in Figure 12a, the red band shows the most apparent peaks among RGB bands. Figure 12b compares peak detections of proposed and conventional methods and shows similar peak detection performance. Figure 13 shows frequency analysis on the signals obtained from our proposed and conventional algorithms. Here, red bands are reassured to have the highest power at the heart rate frequency. We estimated heart rates of ten subjects similarly to Table 1. Estimated heart rates of our proposed and conventional algorithms have the maximum deviation of 0.125 Hz using the time-based estimation method while they have the maximum of 0.03 Hz using the frequency-based estimation method.

## 5. Conclusions

Real-time heart rhythm and rate detection using smart devices has gained much attention. Given that heart rhythm and rate detection has growing interest in the point-of-care health area, more convenient and accurate heart rhythm and rate detection technology is required. In this paper, we have shown that the heart rhythm and rate of healthy subjects can be accurately estimated using the proposed fingertip image-based detection methods with successive fingertip images recorded by the smartphone cameras. Our proposed heart rate detection methods for the smartphone was designed in two ways: (1) a fingertip curve line movement-based detection; and (2) a fingertip image intensity-based detection. We evaluated our heart rate detection methods with an iPhone and the results demonstrated that our proposed methods have provided heart rhythms with high correlation and heart rate with high accuracy, compared to the conventional algorithms. Given the prevalence of smartphones, our proposed heart rate detection methods using smartphones are expected to provide a convenient and accurate way to monitor heart rhythm and rate in our daily lives.

## Figures and Tables

**Table 1:** Mean and standard deviation (STD) heart rate (HR) of ten healthy subjects estimated by the proposed and conventional methods.

Subject	Estimated Mean ± STD HR (Proposed, Time)	Estimated Mean ± STD HR (Conventioal, Time)	Estimated Mean ± STD HR (Proposed, Frequency)	Estimated Mean ± STD HR (Conventioal, Frequency)
1	1.8078 ± 0.1170	1.8128 ± 0.1511	1.7460 ± 0.0716	1.7820 ± 0.0502
2	1.1370 ± 0.0493	1.1451 ± 0.0780	1.2900 ± 0.2683	1.2900 ± 0.3354
3	1.3840 ± 0.0401	1.4014 ± 0.1170	1.4229 ± 0.0596	1.4314 ± 0.1006
4	1.3776 ± 0.0932	1.3847 ± 0.1201	1.3513 ± 0.1649	1.4257 ± 0.2683
5	1.0433 ± 0.1647	1.0475 ± 0.1645	1.0839 ± 0.2937	1.1817 ± 0.3832
6	1.3629 ± 0.1508	1.3779 ± 0.1992	1.3983 ± 0.0880	1.3878 ± 0.0758
7	1.1155 ± 0.1733	1.1066 ± 0.1251	1.1714 ± 0.2439	1.1030 ± 0.1443
8	1.6486 ± 0.2009	1.6331 ± 0.1969	1.6725 ± 0.0521	1.7625 ± 0.0991
9	1.2986 ± 0.1537	1.3419 ± 0.3446	1.3057 ± 0.1682	1.1817 ± 0.4451
10	1.9577 ± 0.0828	2.0409 ± 0.4093	2.0182 ± 0.0911	1.9555 ± 0.2154
